# A Qualitative Evaluation Exploring Co‐Production of Falls Management in Care Homes

**DOI:** 10.1111/hex.70500

**Published:** 2025-11-19

**Authors:** Fran Hallam‐Bowles, Alice Kilby, Adam Gordon, Stephen Timmons, Pip Logan, Lindsay Rees, Will Lawry, CHAFFINCH stakeholder group, Katie Robinson

**Affiliations:** ^1^ Research and Innovation Nottingham University Hospitals NHS Trust Nottingham Nottinghamshire UK; ^2^ Centre for Rehabilitation and Ageing Research, School of Medicine University of Nottingham Nottingham Nottinghamshire UK; ^3^ Nottinghamshire Healthcare NHS Foundation Trust Nottingham Nottinghamshire UK; ^4^ Wolfson Institute of Population Health Queen Mary University of London London UK; ^5^ Academic Centre for Healthy Ageing Barts Health NHS Trust London UK; ^6^ Centre for Health Innovation, Leadership and Learning Nottingham University Business School Nottingham Nottinghamshire UK; ^7^ Surgical Treatment and Rehabilitation Service University of Queensland Brisbane Queensland Australia; ^8^ Avery Healthcare Group Northampton Northamptonshire UK; ^9^ Royal Devon University Healthcare NHS Foundation Trust Exeter Devon UK

**Keywords:** co‐creation, co‐design, falls prevention, long‐term care

## Abstract

**Background:**

Co‐production approaches are increasingly used in research but are rarely evaluated in care home settings. This study explored factors influencing key principles of co‐production in a series of workshops around falls management in care homes.

**Methods:**

Stakeholders (care home residents and relatives, care home staff, and health and social care staff) participating in co‐production workshops as part of a research study were invited to take part in this qualitative evaluation. The workshops were developing a model to implement falls training in care homes as part of a systemic action research study. Non‐participant observations of workshops explored stakeholder interactions. Stakeholders participated in reflection meetings about their experiences of co‐production. Framework analysis mapped key themes to the National Institute for Health and Care Research's (NIHR) principles of co‐production.

**Results:**

Nine themes were identified. Sharing power was affected by two themes: opportunities to challenge dominant voices, resulting from the influence of the research team and separate stakeholder groups, and integrating a disjointed system. Including all perspectives and skills was influenced by two themes: involvement of key stakeholders and a flexible approach. Respecting and valuing knowledge was impacted by two themes: respecting and utilising expertise and experience, and confidence. Two themes relating to reciprocity were identified: benefits and potential harms. One theme related to building and maintaining relationships: team dynamics.

**Conclusions:**

Co‐production in this context is complex and affected by multiple factors. Separate stakeholder groups, a flexible approach and recognising different experiences and expertise facilitated co‐production in line with its key principles. Potential reputational risks, confidence levels and limited involvement of residents, relatives and care home staff in a variety of roles were identified as barriers. Future studies in care homes should consider organisational power dynamics and create safe spaces, providing opportunities for inclusive participation.

**Patient and Public Contribution:**

A collaborator group, including a patient and public involvement and engagement (PPIE) advisor and health and social care professionals, contributed to the research methods, presentation of findings and authorship. Care home residents informed the design of the co‐production workshops.

## 1 Introduction

Institutions providing long‐term care are defined in various ways internationally. In England, care homes are regulated organisations that provide personal care and accommodation, with or without nursing care, currently for around 425,000 adults [1, 2]. Care homes are predominantly privately owned and have diverse characteristics, including their size and quality rating as determined by the statutory regulator [3]. Older adults living in care home settings have complex needs, including dementia and multimorbidity, with consequent high use of services across health and social care (HSC) systems [4]. Their care requires collaborative working between care homes and wider HSC organisations. Co‐production involving care homes and supporting external statutory services has been recommended to improve resident care and to design complex interventions which can be routinely embedded in care homes [5, 6].

Co‐production is a form of collaboration that is increasingly applied in HSC settings [7, 8, 9]. Co‐production is an ambiguous concept that has been variably defined and operationalised, leading to concerns about tokenism and misappropriation of the term [7, 10, 11]. In this study, co‐production was defined as stakeholders working together as equals in their views and participation.

Our conceptualisation of co‐production was based on the National Institute for Health and Care Research's (NIHR) key principles: sharing power, including all perspectives and skills, respecting and valuing knowledge, reciprocity, and building and maintaining relationships [12]. Achieving these principles in care home contexts may be challenging. Difficulties with integrated working between care homes and wider HSC system partners have been noted in previous research, including differences in organisational policies, and a tendency for health organisations to impose ways of working on care homes [13, 14, 15]. Many care homes have limited opportunities to experience research and workforce challenges may affect engagement [16].

While involving residents as experts by experience is noted as a facilitator of collaborative care home research, physical and mental impairments may hinder participation [17, 18]. Ageism and power dynamics influence collaboration with older people and may mean their expertise is undervalued [18, 19]. Few studies have evaluated co‐production approaches in care home settings, including how the co‐production process is experienced by different stakeholders [20]. There is a need to evaluate stakeholder experiences and factors influencing the achievement of key principles of co‐production in care homes to avoid tokenism and inform future research in this context.

## 2 Materials and Methods

This qualitative evaluation aimed to explore how principles of co‐production were experienced and factors influencing their achievement when developing a model for falls management in care homes. The evaluation is reported in accordance with the Standards for Reporting Qualitative Research [21].

Falls are a high‐priority topic because they affect all care homes and HSC systems in the UK and form part of national policy [22]. The evaluation was embedded within the CHAFFINCH study (Co‐producing tHe implementAtion oF Falls management IN Care Homes) [23]. A short summary of the CHAFFINCH study is outlined below to provide context.

### 2.1 Context: The CHAFFINCH Study

CHAFFINCH was a systemic action research study which aimed to co‐produce a model for implementing falls management training in care homes over 12 months. The study was part of a larger programme of research exploring the implementation of the Action Falls programme in care homes [24]. The Action Falls programme, which trains care home staff to use a checklist to identify risk factors and take action to reduce falls among care home residents, demonstrated effectiveness in reducing falls in a large multicentre randomised controlled trial [25].

**FIGURE 1:**
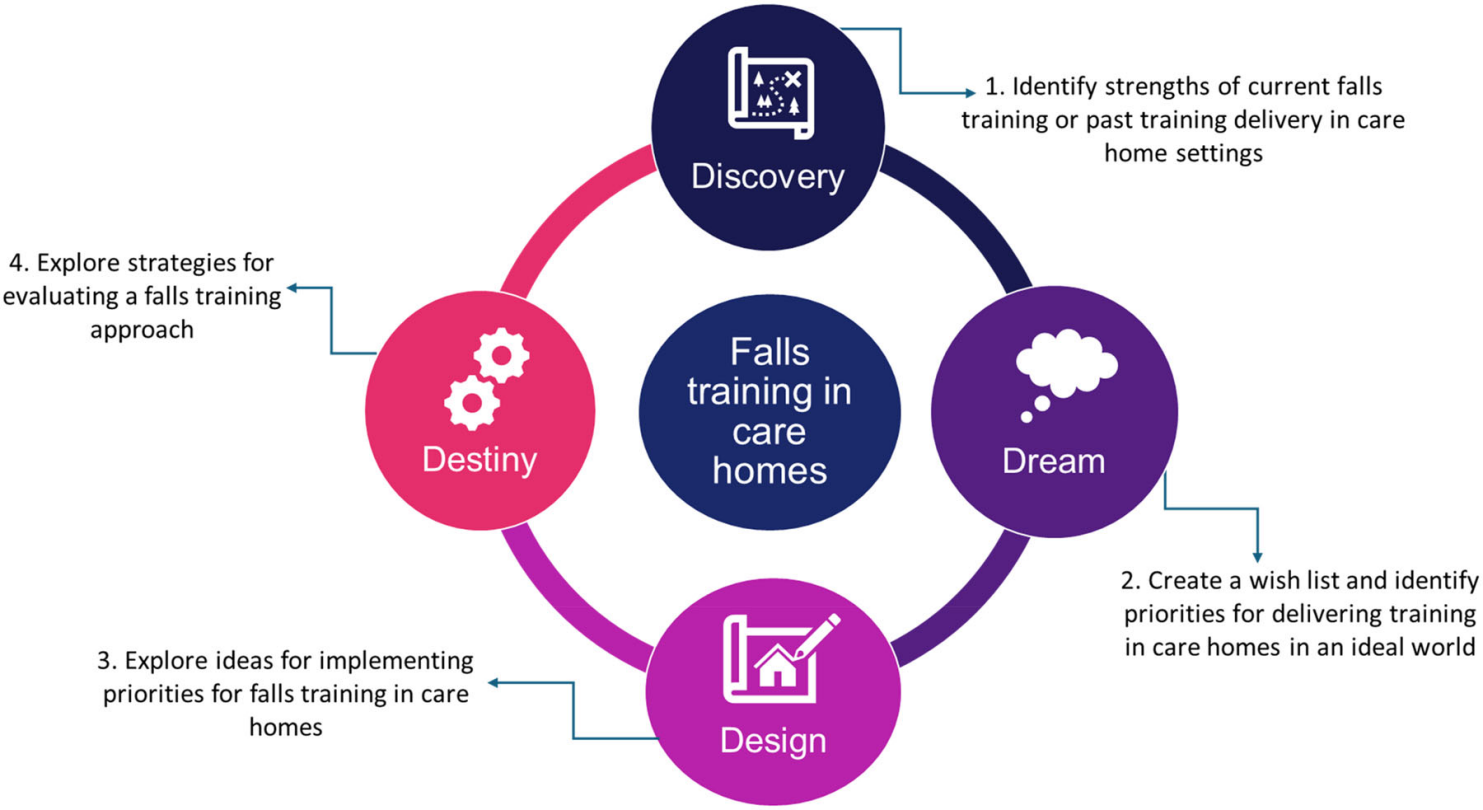
Summary of workshop stages.

Stakeholders participating in the CHAFFINCH study were invited to attend four workshops, followed by a multi‐stakeholder celebration event where the implementation model was finalised. The celebration event was not included in the evaluation as most of the co‐production activities occurred in the co‐production workshops. Workshop participants were stakeholders involved in or affected by falls management in care homes across Nottingham and Nottinghamshire Integrated Care System (ICS). ICSs are statutory place‐based partnerships between HSC organisations that have a responsibility for developing shared plans and delivering coordinated services in England [26]. Information about the Action Falls programme was shared with stakeholders. Workshops followed the 4D stages of Appreciative Inquiry, which is a strength‐based approach for enacting organisational change [27]. A summary of workshop stages and sub‐aims is presented in Figure 1.

Workshops were conducted with three separate stakeholder groups (residents and relatives from one home, care home staff from two homes and HSC organisation representatives). Workshops with care home staff and HSC professionals were online group meetings; whereas workshops with resident and relative stakeholders were one‐to‐one, in‐person conversations at their care home. Workshops were facilitated by a researcher or member of the HSC stakeholder group with experience in facilitating multi‐stakeholder workshops.

### 2.2 Evaluation Design

The qualitative evaluation was conducted between May and November 2023. The evaluation comprised non‐participant observations of workshops and reflection meetings completed after the final workshop.

#### 2.2.1 Non‐Participant Observations

Workshops facilitated by a stakeholder from the HSC representatives group were observed by one researcher (FHB). Using a structured template, field notes were made about organisation of workshops, attendance, topics discussed, decision‐making and stakeholder interactions (see Additional File 1). Stakeholder names were anonymised and identification codes used. Project documents from workshops, including agendas, summaries, reflection forms and picture prompt sheets, were reviewed to corroborate or refute data from observations and interviews.

#### 2.2.2 Reflection Meetings

Reflection meetings were facilitated discussions that explored stakeholders' experiences of the co‐production process. The term ‘reflection meeting’ was selected in line with the reflective component of the action research approach [28]. Meetings were completed individually or in groups, online or in‐person, according to stakeholders' preferences. Meetings lasted up to one hour and were semi‐structured with questions guided by a meeting schedule (see Additional File 2) and insights from early reflections from workshop observations. Meetings were digitally audio‐recorded with consent or notes were made by the researcher. Recordings were transcribed verbatim and anonymised.

### 2.3 Participants

All stakeholders attending co‐production workshops were invited to participate in the evaluation. Each stakeholder provided written or verbal informed consent to take part in the observed workshops and reflection meetings and for direct quotes to be used. The capacity of residents and relatives to provide informed consent was assessed at the start of each meeting. All stakeholders were able to provide their own informed consent.

Eight workshops were observed, including two resident meetings and three meetings each with HSC representatives and care home staff. Thirteen out of sixteen stakeholders attended the observed workshops. This included one care home resident, six care staff, and six HSC organisation representatives from a local authority, a care home organisation and NHS services. The care home resident was an older adult with cognitive impairment and a history of falling. Residents and care staff lived or worked in medium‐sized nursing homes (registered for up to 45–65 people) providing dementia care with a good or outstanding rating from the Care Quality Commission. The Care Quality Commission is the independent regulator of HSC organisations in England and is responsible for registering, monitoring, inspecting and rating care providers [29]. One home was part of a small chain, and two were single owner‐operator homes. Two care homes had previously participated in research.

Nine stakeholders took part in reflection meetings (five HSC representatives, three care staff, one resident). Five stakeholders took part in reflection meetings in small online groups of two to three participants. Four stakeholders participated in individual interviews (two in‐person, two online).

### 2.4 Data Analysis

Data collected from observations and reflection meetings were managed in NVivo 12 and analysed using framework analysis. This approach analyses qualitative data using an a priori theoretical framework and is suited to answering evaluative research questions [30, 31, 32]. The analysis involved five stages, which were interdependent and iterative.

1. Familarisation by listening to recordings and reading data items.
2. An initial thematic framework was identified based on a priori knowledge of the NIHR principles of co‐production and factors affecting their attainment identified in our earlier work [20].
3. The data set was indexed using the framework categories by one researcher (FHB). A second researcher who was not involved in delivering the workshops (KR) independently indexed a selection of the data (one transcript, three field notes). Refinements were made to the framework during the indexing stage. Categories were rearranged into emergent themes.
4. Indexed data was organised and summarised in a charting matrix. Data was separated into cases for each stakeholder group. A separate case was used for the HSC stakeholder who facilitated workshops. Supporting verbatim excerpts were included to maintain a sense of the stakeholders' voice.
5. Patterns across data were mapped by comparing across and within framework components. Themes were defined and linkages identified. Themes were sense‐checked by the research team (KR, ST, PL, AG) and member‐checked by stakeholders.

### 2.5 Reflexivity

The researcher's experiences and relationships shaped data collection and analysis. The researcher who conducted data collection and primary data analysis (FHB) is a physiotherapist with experience working with care homes to manage falls and was a PhD researcher in the team undertaking the CHAFFINCH research programme. FHB had prior working relationships with some stakeholders from care homes and healthcare settings. When not observing, FHB facilitated workshops. Her status as an ‘insider’ and ‘outsider’ to the co‐production process was fluid [33]. FHB's role was explained to stakeholders at the start of each workshop. Regular reflection was undertaken through journalling and discussion with an experienced supervisory team (ST, PL, KR).

### 2.6 Patient and Public Involvement and Engagement

A collaborator group supported the research design, data interpretation and dissemination. The group met online every 3–7 months during the research programme. Group members included a PPIE advisor with past experience as a GP, carer and care home resident, a care home quality and policy lead, a consultant therapist in falls prevention, a care home support service matron, a local authority social care lead, an advanced practitioner and a community falls prevention clinical practitioner. The design of the co‐production workshops was informed by one‐to‐one conversations with care home residents from two care homes. The study information sheet was reviewed by the PPIE advisor. Collaborators are co‐authors on the manuscript where authorship requirements were met.

## 3 Results

**FIGURE 2:**
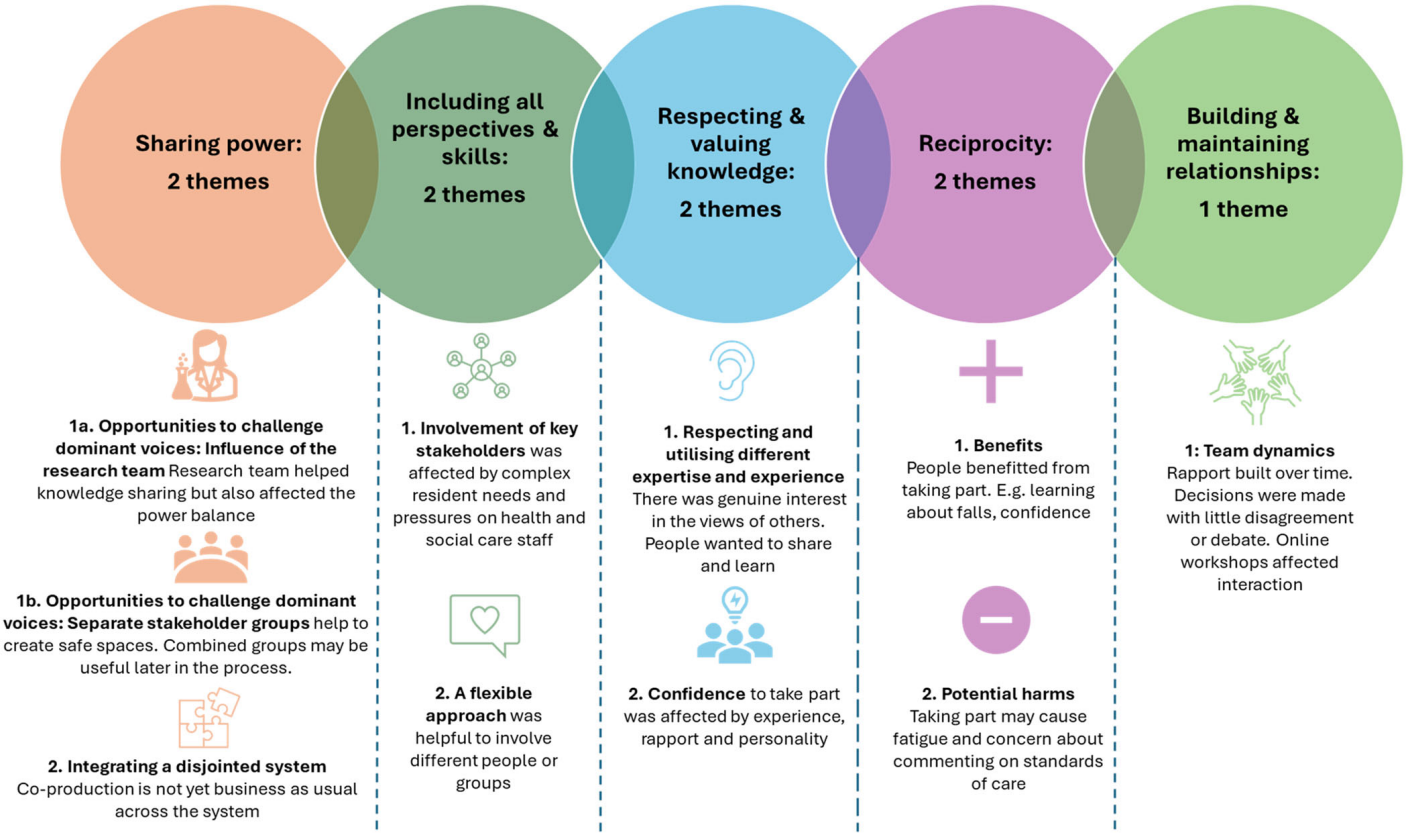
Summary of themes.

Nine themes influencing the achievement of the NIHR principles of co‐production were identified. A summary of themes and their alignment with the principles is shown in Figure 2; however, the themes should be regarded as interconnected.

### 3.1 Sharing Power

Two themes were identified concerning shared ownership of the co‐production process: opportunities to challenge dominant voices and integrating a disjointed system.

#### 3.1.1 Opportunities to Challenge Dominant Voices

The theme had two sub‐themes: the influence of the research team and separate stakeholder groups.

##### 3.1.1.1 The Influence of the Research Team

The influence of the research team refers to all co‐investigators involved in the research design and delivery of the workshops, and all workshop facilitators. The research team gathered, shared and translated views and ideas across stakeholder groups, which appeared to support consideration of different viewpoints. The research team's role was to hold and communicate the stakeholder groups' collective knowledge as this accumulated from previous workshops. This was particularly important for the care home resident with cognitive impairment who struggled to recall details from previous discussions.

‘Telling me what we've talked about before, going over it helps’.

(Reflection meeting, care home resident)

The position of the university as *‘a neutral body’* with less involvement in regulation and commissioning was viewed as beneficial for supporting care home stakeholders to participate in the process by HSC professionals. The research team's previous experience of working with care homes also appeared to encourage participation.

‘They really could say anything in terms of reality for you, whereas I still think they perhaps wouldn't for me’.

(Reflection meeting, HSC representative)

However, project leadership by the research team created challenges for shared ownership. Opportunities for stakeholders to input into the delivery of workshops, such as chairing meetings or suggesting changes to the process, were generally not taken up. However, on one occasion, care staff took an active role in suggesting the need for wider perspectives, collecting this information outside of meetings and feeding it back to the group.

‘Facilitator asks if anyone has ideas for the next workshop. All goes quiet on the call. She asks if anyone else should be involved in the workshops. One stakeholder thinks it would be good to ask carers for their thoughts on ideal training, another suggests the researcher send some questions for them to gather feedback on’.

(Field note, workshop 2 with care home 1)

##### 3.1.1.2 Separate Stakeholder Groups

Separate groups were pivotal for creating safe environments for stakeholders to voice their perspectives. The small group size was reported to facilitate participation by care home staff. Separate groups for residents, relatives, care home staff and HSC representatives, as well as separating care staff and care home management into different groups, helped to disrupt hierarchical power dynamics.

‘I don't think they'd [care staff] have said as much if they'd have been in the same group as me. And so I think that's good because I think sometimes they might have thought, “oh there's a member of management there, what if I say something wrong or something that they don't like?”’

(Reflection meeting, care home representative on the HSC group)

Despite the benefits of separate groups, this approach may have limited opportunities to build more in‐depth, shared understanding between stakeholders. Care home staff and some HSC representatives expressed a preference for multi‐stakeholder groups later in the co‐production process.

‘It's like the saying isn't it, once you've worked in a care home you've always worked in a care home. But it would be nice to hear from how they [health professionals] do things from their side. That kind of gives you then a bit more understanding’.

(Reflection meeting, care home staff)

#### 3.1.2 Integrating a Disjointed System

HSC representatives identified systems‐level influences impacting shared ownership of implementation. It was noted that co‐production was not yet business as usual across the ICS, nor a routine aspect of implementing national directives. There was a shared belief among the HSC representatives that co‐production at a systems‐level would be beneficial for improving resident care.

‘I could put in what I like as a national driver, but how would that help Mrs Jones in room 5, which is the purpose of what we're doing, so I think it's got to be a non‐negotiable way moving forward’.

(Reflection meeting, HSC representatives)

However, HSC stakeholders reported greater investment and culture change at a systems level was needed for a sustainable, embedded approach to co‐production that stretched beyond a single project.

‘I think we probably need to just keep that stakeholder engagement rather than have it peak and drop down again and then build it up again… that's going to take investment, and time as well as monetary investment—that also feels a little bit of a different shift away from commissioning and providers that feels much more collaborative’.

(Reflection meeting, HSC representative and facilitator)

### 3.2 Including All Perspectives and Skills

Including a range of perspectives and skills in the co‐production process was influenced by the extent to which key stakeholders were involved and was facilitated by a flexible approach.

#### 3.2.1 Involvement of Key Stakeholders

There was representation from across the ICS, although the number of residents, relatives and variety of care home staff by role was limited. Attendance of care home staff and HSC representatives at workshops fluctuated and was unpredictable due to the busy nature of their roles and staff turnover in care homes. The online workshop format was challenging at times in busy care environments and using interactive tools was difficult for care staff joining on smartphones. However, care home staff and HSC representatives felt the online workshop format was overall most accessible. This was in terms of minimising travel, working around other commitments and supporting people with learning differences to access information.

‘I've got pure dyslexia. So for me, doing it this way [online] and being able to hear it and see it, it's better for me, than looking at your paper here on the desk’.

(Reflection meeting, care home staff)

Supporting resident involvement in decision‐making about the location of in‐person meetings involved balancing her comfort in a familiar environment against distractions and the level of stimulation. Although the resident engaged in the workshop discussions, cognitive impairment and fatigue affected her articulation of opinions and participation in decision‐making at times.

‘Facilitator asks the resident whether training should focus on prevention or post‐falls management. She seems to be checking whether the resident is just agreeing with her by switching the question around, but it is still unclear if the resident has a strong opinion on this’.

(Field note, resident workshop 2)

#### 3.2.2 Flexible Approach

Adapting workshops to the needs and preferences of groups and individuals was reported as a facilitator for including different perspectives. Flexibility required continuous reflection and questioning of the approach.

‘I feel like having different approaches for the different groups really worked – I feel like we heard their voice’.

(Reflection meeting, HSC representative and facilitator)

A flexible approach required preparation outside of the workshops when planning activities, for instance, creating visual prompts to use in discussions with residents. Skilled facilitation was required in group workshops to *‘keep the conversation going’* (reflection meeting, care home staff). This involved adapting to the interests and energy of individuals, changing the language used, providing encouragement and dealing with logistical challenges. For example, the facilitator adapted the workshop plan to accommodate late arrivals and to focus on topics that sparked interest among stakeholders. She encouraged quieter group members to contribute to discussions. Flexibility was required to weave other perspectives into discussions between groups as opportunities arose. An example is provided in the quote below.

‘Stakeholder reflects that care home staff may have different education levels. Facilitator shares that similar comments have been raised by care home staff about different learning needs in previous discussions and gives some brief details about this’.

(Field note, HSC group workshop 4)

Flexibility was particularly important for facilitating resident involvement. One‐to‐one, in‐person discussions enabled a personalised approach. Using tangible prompts (e.g., falls information resources), mirroring language and weaving questions into reminiscence discussions were strategies used to support resident participation.

‘The facilitator appears to go along with the topic the resident wants to talk about, hears her stories and then weaves questions for discussion into the conversation.’

(Field note, resident workshop 2)

### 3.3 Respecting and Valuing Knowledge

Recognising and using different expertise and experience, along with the confidence levels of stakeholders, contributed to the principle of respecting and valuing knowledge during the co‐production process.

#### 3.3.1 Recognising and Utilising Different Experience and Expertise

Stakeholders contributed different skills and experiences to shape the model for falls management. This included lived experience of falls, insights as the end‐user of falls training, organisational knowledge, and experiences of supporting workforce development or quality improvement in care homes. Differences in perspectives between groups were noted and shared across groups. While all groups seemed interested in hearing other perspectives, the HSC group reflected and incorporated these into their reasoning more than the other stakeholder groups.

‘Discussion about outcome suggestions made by other groups. One stakeholder thinks the resident and relative suggestions pick up more on falls prevention, while care home staff responses suggest a more reactive rather than proactive approach. The group discusses whether care home staff realise their own power and identify confidence as important’.

(Field note, HSC workshop 4)

Workshops were described as non‐judgemental spaces. Stakeholders commented on feeling equal regardless of their role, seniority or organisation. Feeling listened to seemed particularly important for supporting the participation of those working in care homes. Attributes of the individuals involved helped to create a respectful environment. For instance, stakeholders had a desire to learn and share knowledge, were genuinely curious about other opinions and had a passion for improving future care.

‘Nobody's come across as if they're better than anybody else’.

(Reflection meeting, care home staff)

#### 3.3.2 Confidence

Confidence to participate in the co‐production process was affected by stakeholders' familiarity with co‐production activities and how these fitted with their role. Care staff seemed less familiar with co‐production and participating in online workshops as a part of their role, whereas HSC representatives in leadership positions viewed participation as a routine, priority activity. Confidence of care staff and HSC representatives also seemed to vary at different stages of the Appreciative Inquiry cycle [27]. Care staff appeared more confident with ‘dreaming’ priorities for falls training, whereas HSC representatives caveated their blue sky thinking with experiences of reality. In contrast, HSC representatives seemed more confident with suggesting implementation and evaluation strategies as they had previous experiences to draw upon. It was suggested that involvement in strategic decision‐making may be at odds with traditional organisational practices in care home settings.

‘“what do you think – shall we do it like this – how shall we do that?” Probably feels quite new. I think care home staff have probably had years and years of, “go and do this and fill in this form and do it this way – now off you go.” So I think it's probably a new concept and that cultural shift of working like that is probably quite challenging’.

(Reflection meeting, HSC representative and facilitator)

Confidence increased over time through developing rapport with facilitators and group members. The structure of the workshops, including small groups, clear objectives and one‐to‐one discussions were reported facilitators. Some stakeholders reported that personality differences influenced confidence in group scenarios.

‘I am confident in myself to talk in a large group. I am who I am’.

(Reflection meeting, care home staff)

### 3.4 Reciprocity

Benefits from taking part in co‐production and potential harms were identified during the process.

#### 3.4.1 Benefits

All stakeholders reported benefiting from participation in the co‐production process. Short‐term benefits included a sense of fulfilment from helping others, learning about falls management, accessing falls training resources, connecting and reflecting on ways of working.

‘I like to be helpful. This care home has a good reputation, it's good to help other care homes’.

(Reflection meeting, resident)

For care homes, there were wider benefits in raising confidence and awareness of falls management among participating staff who then shared knowledge with colleagues.

‘It definitely gained my confidence and I think for the care staff that took part as well, I can definitely see a change in them and they are already being more aware about the things they need to be looking out for a bit more and speaking to other staff’.

(Reflection meeting, care home representative on the HSC group)

Opportunities to continue learning together and building capacity for co‐production across the system were noted as potential longer‐term benefits. HSC representatives highlighted the importance of selling the benefits of co‐production and demonstrating tangible change to encourage care homes to be involved in future work. HSC representatives described a heightened understanding of ‘genuine’ co‐production and its time requirements.

‘I think it's given me a really good questioning brain into when people say, “oh this has been co‐produced”’, I want to go, “tell me about how you co‐produced that”. I think co‐production can be a bit of a flashy word to use at the minute and so was this genuine co‐production or did you just ask some people to comment on it? It's given me that greater curiosity into that co‐production process and a much better understanding of if we're going to do genuine co‐production, just how long that takes to get to a point where you are all feeling of equal value in opinions’.

(Reflection meeting, HSC representative and facilitator)

#### 3.4.2 Potential Harms

Disadvantages of co‐production were rarely reported by stakeholders, although possible harms were noted. There was a risk of fatigue from participation in online meetings. Interacting with the facilitator and falls information resources at times appeared draining for the care home resident, leading to cognitive overload and fatigue.

‘Facilitator begins summing up key points. Resident closes her eyes again and seems very tired suddenly. Facilitator makes the decision to stop the discussion’.

(Field note, resident workshop 3)

There also appeared to be concerns about potential repercussions arising from commenting on standards of care. The resident involved in this process indicated that residents' feelings about care quality may influence their confidence to share their opinions openly.

‘Researcher: How confident did you feel to say your thoughts?

Resident: Not sure. The staff are very good here but I know not all care homes are the same, I'm not sure if it would be different somewhere else’.

(Reflection meeting with resident)

HSC representatives were mindful of past negative experiences of collaborating to manage falls in care homes. Care home staff participating in their own group and the HSC representatives group seemed conscious about maintaining a positive image of their care home.

‘I was representing my whole care home so what if I said something wrong and then someone's like “oh what have you said that for?”’

(Reflection meeting, care home representative on the HSC group)

### 3.5 Building and Maintaining Relationships

#### 3.5.1 Team Dynamics

Rapport between group members developed over time through interaction during workshop activities.

‘I think just throw everybody on the internet together and let them talk’.

(Reflection meeting, care home staff)

Each group tended to agree and bounce ideas off each other when making decisions. Although agreement appeared genuine, it was difficult to unpick whether stakeholders were reluctant to share different opinions.

‘I don't think we've come across work conflicts at all – but if we did have a difference of opinion I'm sure I've felt confident that we could tackle it professionally’.

(Reflection meeting, HSC representative)

Although the online format had benefits for accessibility, it also impeded interaction and establishing relationships.

‘it seemed a bit lagging, I think it's harder to tell whether somebody actually wants to speak or not, because you don't know if you're going to be interrupting someone, whereas I think when you're face to face in a group you can kind of judge it or you can directly look at that person’.

(Reflection meeting, care home representative on the HSC group)

### 3.6 Recommendations

Table 1 outlines recommendations for co‐production in care home research in accordance with the NIHR's principles. The recommendations are based on the themes reported in this evaluation and our cumulative findings during this study programme. Recommendations were developed with a study collaborator group member with experience of working in care homes and a stakeholder from a care home organisation who participated in this study.

**TABLE 1:** Recommendations for co‐production in care homes.

Principle	Recommendation
Sharing power	Plan and continually reflect on organisational power dynamics that affect how stakeholders work together. Consider how: Organisational culture in care homes, wider HSC and research organisations impacts stakeholder participation Regulation and policy drivers shape collaboration between care home and HSC organisations (e.g., local and Care Quality Commission quality assurance monitoring requirements) Stakeholders perceive the organisation leading the project Research governance processes affect power sharing. Discuss their implications with research sponsors and ethical review panels.
	Creating safe spaces supports participation in co‐production in care homes. Consider: The timing of separate and mixed stakeholder groups Acceptable ways of working that account for stakeholder preferences How researchers and facilitators can assist less dominant voices to be heard by acting as knowledge brokers. This requires research teams to be reflexive and adaptable.
	Co‐production across HSC systems requires resource investment and culture change.
Including all perspectives and skills	Work with stakeholders, PPIE members and collaborators to plan accessible co‐production activities. Consider using: Personalised approaches to support residents with complex needs to take part. Consult families and care home staff as appropriate Accessible information and creative approaches to support resident and relative involvement Practical approaches that can be embedded into busy care home environments
	Build flexibility into project plans. Work with stakeholders to adapt the co‐production process according to their strengths, needs and preferences, as resources allow. For instance, consider the accessibility of online and face‐to‐face activities and scheduling meetings to include staff with different roles and working patterns.
	The availability of key stakeholders can lead to trade‐offs between key principles of co‐production and what is practically possible. Report the impact of these challenges and any strategies used to overcome them to help inform future projects.
Respecting and valuing knowledge	Consider the values, motivations and problems of stakeholders. Plan activities around these to promote genuine interest.
	Support care home residents, relatives and staff to build confidence and experience in co‐production. Possible strategies include: Using a structured approach Working with stakeholders to assess how the proposed co‐production activities align with their experiences, skills and roles, and identify which aspects of co‐production they would like to be involved in Providing training and support to bridge any gaps between aspects of co‐production the individual would like to be involved in and their current knowledge/skills (as workload allows)
Building and maintaining relationships	Time is required to build relationships, shared understanding and trust between stakeholders.
	Establish regular communication channels, ways of working and a shared definition of co‐production.
	Approaches that utilise existing relationships between care home staff, such as group activities, may be beneficial. For example, incorporating co‐production activities into existing meetings (handovers, team meetings).
	Management support is important for care homes to participate in co‐production; however, maintaining this relationship can be challenging.
Reciprocity	Clearly outline the potential risks and benefits of taking part for each stakeholder group. Time is required to build trust and alleviate concerns.
	Evaluation can support the identification of risks and benefits to aid planning of future research.
	Consider how to keep involving stakeholders and demonstrate progress in the later stages of co‐production.
Other: Logistical arrangements	When planning co‐production in care homes, consider: The availability of time, resources, funding and a skilled and experienced team How care home environments might affect co‐production activities (e.g., availability of private spaces for workshops or interviews)

*Note:* [Bold text identifies row and column headings]

## 4 Discussion

This evaluation explored how principles of co‐production were experienced and factors influencing their achievement when developing an model for implementing falls training in care homes across a HSC system. The co‐production process was overall a positive experience for stakeholders which resulted in benefits for individuals and organisations. The findings highlight that co‐production in this context is complex and affected by a range of factors. These include characteristics of individuals (such as roles, impairments and confidence), interpersonal relationships, organisational pressures and priorities, and system influences. A flexible approach and creating safe spaces facilitated co‐production. A one‐to‐one, personalised approach with skilled facilitation was beneficial for supporting resident participation. Our findings suggest that co‐production in this setting requires investment in resources and consideration of how organisational dynamics across the system interact when brought together.

### 4.1 Wider Context

In alignment with previous research, this evaluation highlights that safe communicative spaces are important for optimising communication between researchers and care home residents acting as collaborators in research [18]. However, a novel finding of this study is that safe spaces are also important for equalising power between care home staff and HSC professionals both within and across organisations. The evaluation demonstrates this approach is particularly suited to neutralising power dynamics between care home staff in management and frontline care roles. Previous research shows organisational culture in care homes varies [34] and this needs to be considered when deciding when and how to separate care home staff to optimise stakeholder participation in the co‐production process. However, while separate groups were useful for equalising power, they limit opportunities to build relationships across stakeholder groups. Multi‐stakeholder groups have been reported to support connection and shared understanding in previous co‐production literature in care homes [35, 36]. Blended approaches may be required to support participation based on the needs of stakeholders, each with different trade‐offs for achieving all key principles of co‐production, and this is an area that warrants further research.

Another interesting insight from this evaluation is that participation in co‐production can introduce reputational risks for care home organisations within a system of regulation. Risks from an organisational or professional perspective are often under‐acknowledged in the existing co‐production literature [37]. Previous patient safety research in care homes suggests that regulation inadvertently contributes to blame cultures within care homes and across the system, which can negatively affect team cohesion and limit opportunities for staff to be involved in quality improvement processes [38, 39, 40]. The expertise of care home staff can be de‐valued and collective responsibility becomes challenging when there is a high level of focus on compliance [38, 41, 42]. These conditions are at odds with key principles of co‐production, such as respecting different forms of knowledge and sharing power. Previous experiences of cross‐organisational collaboration in a system of regulation can affect the confidence of care home staff involved in co‐production. A key finding from this evaluation is that co‐production may feel uncomfortable for care home staff; therefore, time needs to be invested to build trust and confidence. Time is required to learn about the individual, their organisation, pressures and potential reputational risks. This learning should inform the planning of inclusive co‐production activities which aim to build confidence and relationships.

This study also highlights the ethical and practical challenges of balancing the involvement of residents with cognitive impairment in co‐production against the risk of causing potential harms, such as fatigue, cognitive overload or anxiety. Impairments, low energy levels and apprehension are known barriers for involving residents in PPIE activities [17, 18]. This evaluation extends our knowledge by illustrating how busy care home environments can exacerbate impairments during co‐production activities. While communal areas may be familiar and comfortable, which can support involvement of disadvantaged groups, they can also result in challenges with maintaining confidentiality and supporting people living with dementia to participate in a distraction‐free environment [43, 44]. Clear information about potential risks is required to help residents and their consultees (if required) to make informed decisions about participation. Researchers should involve residents, their families and care home staff who know residents well to mitigate against risks and monitor for signs of distress or fatigue during co‐production activities. The needs and preferences of residents may change therefore flexibility is required.

Our finding that a flexible approach is vital for co‐production in care homes replicates previous findings about supporting PPIE and collaboration with care homes, older people and disadvantaged groups [17, 18, 45, 46]. This evaluation builds on these findings by highlighting the value of personalised and creative approaches, such as reminiscence and one‐to‐one conversations, when supporting residents with cognitive impairments to participate in co‐production. The evaluation provides a practical example of how a flexible approach can be supported. Talking and sharing life stories, using visual prompts and tacit objects align with previous calls to move beyond text‐based approaches to co‐production [47]. Creative methods are beneficial for redistributing power when involving people with lived experience in research [48]. Older people with dementia and carers can contribute important insights to the development of innovative methods [49]. Research teams should work with these groups to further explore acceptable and novel co‐production approaches that allow for creativity and flexibility. Changes to research funding and governance infrastructure may be required to achieve a greater move towards resource‐intensive, iterative methods, which can be directed by stakeholders [47, 50]. Further debate and consultation are required to determine where reform to research governance structures is needed.

Exploration of co‐production over a longer period would be beneficial to aid understanding of the effect of group cohesion and conflict on achieving key principles. There are several possible explanations for the lack of disagreement observed in workshops. While agreement seemed genuine, academics inevitably have different values, motivations and approaches to other stakeholders, and these can unintentionally take precedence [37]. This can create challenges for sharing power. Lack of disagreement could suggest that the co‐production groups were still forming and had not reached the later stages of group formation required to achieve cohesion and effective collaboration [51]. Others suggest that a focus on partnership working overlooks the benefits of conflict in neutralising power dynamics [52]. For instance, difficult conversations can indicate that conditions have been created for honest contributions and acknowledgement of past wrongdoings to less dominant groups [53]. Established partnership approaches between academia and care homes, such as the Living Lab model used in the Netherlands and the UK, have the potential to support greater stakeholder involvement in co‐production by redistributing power away from academic institutions towards end‐users of research and supporting co‐production over a longer period through an established network [54, 55]. These partnerships are a useful avenue for evaluating co‐production over a sustained period of time.

### 4.2 Limitations

No relatives consented to participate in the reflection meetings and no relative meetings were observed. The inclusion of resident and relative perspectives in the findings is limited and further research is needed to explore strategies to support their inclusion in future co‐production projects. Stakeholders who did not participate in the reflection meetings may have experienced co‐production differently. This was not an independent evaluation and the findings presented are influenced by the research team's interpretation of the co‐production process and interactions with stakeholders. The evaluation focussed on a snapshot of a specific point in the co‐production process within an established ICS. The findings may not be generalisable to longer co‐production processes or areas where ICSs are developing.

## 5 Conclusions

This study evaluated how principles of co‐production were experienced and factors influencing their achievement when developing a model for implementing falls management across a HSC system. Co‐production was largely a positive experience for stakeholders in care homes. Achieving the principles of co‐production was influenced by complex and interconnected factors. Separate stakeholder groups, adopting a flexible approach, and recognising different experiences and expertise were identified as facilitators. Challenges included potential risks, confidence levels and limited involvement of residents, relatives and care homes staff in a variety of roles. Co‐production approaches used in future research in care homes should consider organisational power dynamics to create safe spaces for participation. Potential risks and benefits of engaging in the co‐production process should be clearly explained. A flexible and personalised approach is required to support the inclusion of key stakeholders in care home settings, including care home residents and staff.

## Author Contributions

This study was undertaken as part of Fran Hallam‐Bowles's doctoral studies. **Fran Hallam‐Bowles:** conceptualisation, methodology, investigation, formal analysis, visualisation, project administration, writing – original draft, writing – reviewing and editing. **Alice Kilby:** methodology, visualisation, writing – reviewing and editing. **Adam Gordon:** conceptualisation, methodology, visualisation, writing – reviewing and editing, **Stephen Timmons:** conceptualisation, methodology, supervision, visualisation, writing – reviewing and editing. **Pip Logan:** conceptualisation, methodology, investigation, supervision, visualisation, writing – reviewing and editing. **Lindsay Rees:** methodology, visualisation, writing – reviewing and editing. **Will Lawry:** methodology, visualisation, writing – reviewing and editing. **CHAFFINCH Stakeholder Group:** validation, visualisation, writing – reviewing and editing. **Katie Robinson:** conceptualisation, methodology, investigation, formal analysis, supervision, funding acquisition, visualisation, project administration, writing original draft, writing reviewing and editing.

## Ethics Statement

The study was given a favourable opinion by the Social Care Research Ethics Committee and received Health Research Authority Approval on the 28th June 2022 (22/IEC08/0015). Written informed consent was obtained for all participants. Written informed consent was obtained to publish the names of individuals in the acknowledgements section.

## Conflicts of Interest

K.R. was a researcher on the Falls in Care Homes (FinCH) trial, where Action Falls was evaluated, P.L. was the CI, and A.G. was a co‐applicant. P.L. is the CI on the Finch‐IMP study, and PL and KR are co‐applicants.

## Supporting information

Additional file 1_Field note template.

Additional file 2_ Reflection meeting schedule.

## Data Availability

The data analysis spreadsheet is available from the corresponding author on reasonable request.

## References

[1] Competitions and Markets Authority . 2017. Care Homes Market Study: Summary of Final Report. [Online], accessed November 1, 2024, https://www.gov.uk/government/publications/care-homes-market-study-summary-of-final-report/care-homes-market-study-summary-of-final-report.

[2] Office for National Statistics . 2023b. *Older People Living in Care Homes in 2021 and Changes Since 2011* [Online], accessed September 10, 2024, https://www.ons.gov.uk/peoplepopulationandcommunity/birthsdeathsandmarriages/ageing/articles/olderpeoplelivingincarehomesin2021andchangessince2011/2023-10-09.

[3] Office for National Statistics . 2023a. *Care Homes and Estimating the Self‐Funding Population, England: 2022 to 2023* [Online], accessed August 13, 2024, https://www.ons.gov.uk/peoplepopulationandcommunity/healthandsocialcare/socialcare/articles/carehomesandestimatingtheselffundingpopulationengland/2022to2023.

[4] A. L. Gordon , M. Franklin , L. Bradshaw , P. Logan , R. Elliott , and J. R. F. Gladman , “Health Status of UK Care Home Residents: A Cohort Study,” Age and Ageing 43 (2013): 97–103. 23864424 10.1093/ageing/aft077 PMC3861334

[5] C. Goodman , S. L. Davies , A. L. Gordon , et al., “Optimal NHS Service Delivery to Care Homes: A Realist Evaluation of the Features and Mechanisms That Support Effective Working for the Continuing Care of Older People in Residential Settings,” Health Services and Delivery Research 5 (2017): 1–204. 29091374

[6] G. Peryer , S. Kelly , J. Blake , et al., “Contextual Factors Influencing Complex Intervention Research Processes in Care Homes: A Systematic Review and Framework Synthesis,” Age and Ageing 51 (2022): afac014. 35231097 10.1093/ageing/afac014 PMC8887840

[7] F. Fusco , M. Marsilio , and C. Guglielmetti , “Co‐Production in Health Policy and Management: A Comprehensive Bibliometric Review,” BMC Health Services Research 20 (2020): 504. 32503522 10.1186/s12913-020-05241-2 PMC7275357

[8] P. Slattery , A. K. Saeri , and P. Bragge , “Research Co‐Design in Health: A Rapid Overview of Reviews,” Health Research Policy and Systems 18 (2020): 17. 32046728 10.1186/s12961-020-0528-9 PMC7014755

[9] H. Smith , L. Budworth , C. Grindey , et al., “Co‐Production Practice and Future Research Priorities in United Kingdom‐Funded Applied Health Research: A Scoping Review,” Health Research Policy and Systems 20 (2022): 36. 35366898 10.1186/s12961-022-00838-x PMC8976994

[10] A. Filipe , A. Renedo , and C. Marston , “The Co‐Production of What? Knowledge, Values, and Social Relations in Health Care,” PLoS Biology 15 (2017): e2001403. 28467412 10.1371/journal.pbio.2001403 PMC5414996

[11] J. Bandola‐Gill , M. Arthur , and R. I. Leng , “What Is Co‐Production? Conceptualising and Understanding Co‐Production of Knowledge and Policy Across Different Theoretical Perspectives,” Evidence & Policy 19 (2023): 275–298.

[12] G. Hickey , S. Denegri , G. Green , et al., Guidance on Co‐Producing a Research project (NIHR INVOLVE, 2018).

[13] H. Gage , A. Dickinson , C. Victor , et al., “Integrated Working Between Residential Care Homes and Primary Care: A Survey of Care Homes in England,” BMC Geriatrics 12 (2012): 71. 23151009 10.1186/1471-2318-12-71 PMC3534387

[14] N. Steils , K. Samsi , and J. Moriarty , The Factors Supporting the Retention of Registered Nurses in Adult Social Care (NIHR Policy Research Unit in Health and Social Care Workforce, The Policy Institute, King's College London, 2024).

[15] R. Stocker , C. Bamford , K. Brittain , et al., “Care Home Services at the Vanguard: A Qualitative Study Exploring Stakeholder Views on the Development and Evaluation of Novel, Integrated Approaches to Enhancing Healthcare in Care Homes,” BMJ Open 8 (2018): e017419. 10.1136/bmjopen-2017-017419 PMC5875673 29581198

[16] E. Law , R. Ashworth , L. Killin , and P. Connelly , “Motivating and Constraining Factors for Research Participation in Scottish Care Homes,” Nursing and Residential Care 23 (2021): 1–7.

[17] T. Backhouse , A. Kenkmann , K. Lane , B. Penhale , F. Poland , and A. Killett , “Older Care‐Home Residents as Collaborators or Advisors in Research: A Systematic Review,” Age and Ageing 45 (2016): 337–345. 26790454 10.1093/ageing/afv201 PMC4846791

[18] T. Burgher , V. Shepherd , and C. Nollett , “Effective Approaches to Public Involvement in Care Home Research: A Systematic Review and Narrative Synthesis,” Research Involvement and Engagement 9 (2023): 38. 37268986 10.1186/s40900-023-00453-2 PMC10234794

[19] J. Bindels , V. Baur , K. Cox , S. Heijing , and T. Abma , “Older People as Co‐Researchers: A Collaborative Journey,” Ageing and Society 34 (2014): 951–973.

[20] F. V. Hallam‐Bowles , P. A. Logan , S. Timmons , and K. R. Robinson , “Approaches to Co‐Production of Research in Care Homes: A Scoping Review,” Research Involvement and Engagement 8 (2022): 74. 36550509 10.1186/s40900-022-00408-z PMC9780102

[21] B. C. O'brien , I. B. Harris , T. J. Beckman , D. A. Reed , and D. A. Cook , “Standards for Reporting Qualitative Research: A Synthesis of Recommendations,” Academic Medicine 89 (2014): 1245–1251. 24979285 10.1097/ACM.0000000000000388

[22] NHS England , *Enhanced Health in Care Homes Framework*. [Online] (London: NHS England, accessed November 1, 2024, 2023), https://www.england.nhs.uk/publication/enhanced-health-in-care-homes-framework/.

[23] Nottingham University Hospitals NHS Trust . n.d. *CHAFFINCH* [Online], accessed November 4, 2024, https://www.nuh.nhs.uk/chaffinch-/.

[24] University of Nottingham . 2024. Community Rehabilitation. Falls in Care Homes (FinCH) [Online], accessed May 2, 2024, https://www.nottingham.ac.uk/Research/Groups/CommunityRehabilitation/Projects/Falls-in-Care-Homes-FinCH.aspx.

[25] P. A. Logan , J. C. Horne , J. R. F. Gladman , et al., “Multifactorial Falls Prevention Programme Compared With Usual Care in UK Care Homes for Older People: Multicentre Cluster Randomised Controlled Trial With Economic Evaluation,” BMJ 375 (2021): e066991. 34876412 10.1136/bmj-2021-066991 PMC8649897

[26] NHS England . n.d. *What Are Integrated Care Systems?* [Online], accessed August 7, 2024, https://www.england.nhs.uk/integratedcare/what-is-integrated-care/.

[27] J. D. Ludema and R. E. Fry , “The Practice of Appreciative Inquiry,” in The SAGE Handbook of Action Research: Participative Inquiry and Practice, eds. P. REASON and H. BRADBURY (SAGE, 2008).

[28] E. T. Stringer , Action Research (SAGE, 2021).

[29] Care Quality Commission . 2022. *Our Purpose and Role* [Online], accessed August 19, 2025, https://www.cqc.org.uk/about-us/our-purpose-role/who-we-are.

[30] S. Parkinson , V. Eatough , J. Holmes , E. Stapley , and N. Midgley , “Framework Analysis: A Worked Example of a Study Exploring Young People's Experiences of Depression,” Qualitative Research in Psychology 13 (2016): 109–129.

[31] J. Ritchie and L. Spencer , “Qualitative Data Analysis for Applied Policy Research.” Analyzing Qualitative Data (Routledge, 1994). 1st ed..

[32] J. Ritchie and L. Spencer , The Qualitative Researcher's Companion (SAGE, 2002).

[33] K. Woodward , “Hanging out and Hanging About: Insider/Outsider Research in the Sport of Boxing,” Ethnography 9 (2008): 536–560.

[34] A. Killett , D. Burns , F. Kelly , et al., “Digging Deep: How Organisational Culture Affects Care Home Residents' Experiences,” Ageing and Society 36 (2016): 160–188.

[35] J. Dugstad , T. Eide , E. R. Nilsen , and H. Eide , “Towards Successful Digital Transformation Through Co‐Creation: A Longitudinal Study of a Four‐Year Implementation of Digital Monitoring Technology in Residential Care for Persons With Dementia,” BMC Health Services Research 19 (2019): 366. 31182093 10.1186/s12913-019-4191-1 PMC6558683

[36] P. Willis , K. Almack , T. Hafford‐Letchfield , P. Simpson , B. Billings , and N. Mall , “Turning the Co‐Production Corner: Methodological Reflections From an Action Research Project to Promote Lgbt Inclusion in Care Homes for Older People,” International Journal of Environmental Research and Public Health 15 (2018): 695. 29642460 10.3390/ijerph15040695 PMC5923737

[37] M. Flinders , M. Wood , and M. Cunningham , “The Politics of Co‐Production: Risks, Limits and Pollution,” Evidence & Policy 12 (2016): 261–279.

[38] K. Abrahamson , R. Fox , A. Roundtree , and K. Farris , “Nursing Assistants' Perceptions of Their Role in the Resident Experience,” Nursing & Health Sciences 22 (2020): 72–81. 31617313 10.1111/nhs.12649

[39] C. Kirkpatrick and B. Nyatanga , “Exploring Perceptions and Approaches of Registered Managers Regarding Clinical Safety in Care Homes in the UK,” Journal of Long Term Care (2023): 45–53.

[40] J. Scott‐Cawiezell , A. Vogelsmeier , C. Mckenney , M. Rantz , L. Hicks , and D. Zellmer , “Moving From a Culture of Blame to a Culture of Safety in the Nursing Home Setting,” Nursing Forum 41 (2006): 133–140. 16879148 10.1111/j.1744-6198.2006.00049.x

[41] A. Banerjee , H. Armstrong , P. Armstrong , et al., “Regulation and Accountability in the Care Home Sector: Expert Commentaries.” Care Homes in a Turbulent Era (Edward Elgar Publishing, 2023).

[42] S. Biggs and A. Carr , “How Provider Organisations Interpret Regulation in the Context of Residential Dementia Aged Care,” Australasian Journal on Ageing 38 (2019): 83–89. 31496058 10.1111/ajag.12634

[43] N. Daniels , P. Gillen , K. Casson , and I. Wilson , “STEER: Factors to Consider When Designing Online Focus Groups Using Audiovisual Technology in Health Research,” International Journal of Qualitative Methods 18 (2019).

[44] K. Samsi and J. Manthorpe , Interviewing People Living With Dementia in Social Care Research (2020) [Online], https://sscr.nihr.ac.uk/wp-content/uploads/2025/04/M022-SSCR-methods-review_MR022.pdf.

[45] F. Cowdell , J. Dyson , M. Sykes , R. Dam , and R. Pendleton , “How and How Well Have Older People Been Engaged in Healthcare Intervention Design, Development or Delivery Using Co‐Methodologies: A Scoping Review With Narrative Summary,” Health & Social Care in the Community 30 (2022): 776–798. 33103313 10.1111/hsc.13199

[46] J. Dawes , D. S. Barron , and L. E. Lee , “Capturing Learning From Public Involvement With People Experiencing Homelessness to Help Shape New Physiotherapy Research: Utilizing a Reflective Model With an Under‐Served, Vulnerable Population,” Health Expectations 25 (2022): 2203–2212. 34891222 10.1111/hex.13397 PMC9615046

[47] Y. Beebeejaun , C. Durose , J. Rees , J. Richardson , and L. Richardson , “‘Beyond Text’: Exploring Ethos and Method in Co‐Producing Research With Communities,” Community Development Journal 49 (2013): 37–53.

[48] O. R. Phillips , C. Harries , J. Leonardi‐Bee , et al., “What Are the Strengths and Limitations to Utilising Creative Methods in Public and Patient Involvement in Health and Social Care Research? A Qualitative Systematic Review,” Research Involvement and Engagement 10 (2024): 48. 38741156 10.1186/s40900-024-00580-4 PMC11092192

[49] L. Phillipson and A. Hammond , “More Than Talking: A Scoping Review of Innovative Approaches to Qualitative Research Involving People With Dementia,” International Journal of Qualitative Methods 17 (2018): 1609406918782784.

[50] B. Smith , O. Williams , L. Bone , and M. S. W. C. Collective , “Co‐Production: A Resource to Guide Co‐Producing Research in the Sport, Exercise, and Health Sciences,” Qualitative Research in Sport, Exercise and Health 15 (2023): 159–187.

[51] D. A. Bonebright , “40 Years of Storming: A Historical Review of Tuckman's Model of Small Group Development,” Human Resource Development International 13 (2010): 111–120.

[52] M. Farr , “Power Dynamics and Collaborative Mechanisms in Co‐Production and Co‐Design Processes,” Critical Social Policy 38 (2018): 623–644.

[53] J. D. Worsley , M. Mckeown , T. Wilson , and R. Corcoran , “A Qualitative Evaluation of Coproduction of Research: ‘If You Do It Properly, You Will Get Turbulence’,” Health Expectations 25 (2022): 2034–2042. 33949751 10.1111/hex.13261 PMC9615072

[54] University of Leeds . n.d. Niche‐Leeds Research [Online], accessed September 4, 2024, https://niche.leeds.ac.uk/niche-leeds-research/.

[55] H. Verbeek , S. M. G. Zwakhalen , J. M. G. A. Schols , G. I. J. M. Kempen , and J. P. H. Hamers , “The Living Lab in Ageing and Long‐Term Care: A Sustainable Model for Translational Research Improving Quality of Life, Quality of Care and Quality of Work,” Journal of Nutrition, Health and Aging 24 (2020): 43–47. 10.1007/s12603-019-1288-5 PMC6934630 31886807

