# Supplementary material for: A Qualitative Evaluation Exploring Co‐Production of Falls Management in Care Homes
Source: Health Expect. 2025 Nov 19;28(6):e70500. doi: 10.1111/hex.70500 (PMC12630552; doi:10.1111/hex.70500)
Supplement: Supplementary file 1 — Additional file 1_Field note template. [file HEX-28-e70500-s001.docx]

**Additional file 1: Field note template**

| **Observer:**  **Date and time:**  **Workshop reference:**  **Workshop format:** | |
| --- | --- |
| **Attendees**  *e.g., Roles, apologies* | **Reflections** |
| **Content and structure of workshops**  *e.g., Agenda, activities, discussion topics, organisation* |  |
| **Stakeholder participation**  *e.g., communication between attendees, conflicts/tensions, decision making, are stakeholder suggestions actioned* |  |
