# Supplementary material for: A Qualitative Evaluation Exploring Co‐Production of Falls Management in Care Homes
Source: Health Expect. 2025 Nov 19;28(6):e70500. doi: 10.1111/hex.70500 (PMC12630552; doi:10.1111/hex.70500)
Supplement: Supplementary file 2 — Additional file 2_ Reflection meeting schedule. [file HEX-28-e70500-s002.docx]

**Additional file 2: Reflection meeting schedule**

**Schedule**

All stakeholders involved in the co-production of a new model for falls management in care homes across Nottingham and Nottinghamshire will be invited to take part in a group reflection meeting. There are expected to be up to 12 stakeholders divided into three groups:

- ICS stakeholder representatives: this may include managers and owners/executives from care homes, commissioners, health care professionals and regulators involved in care home and falls management pathways
- residents living in care homes and relatives of residents living in one care home
- Care home staff working in one care home

One meeting will be conducted for each stakeholder group. The meetings will be managed to last no longer than one hour. Stakeholders will be given the option of attending a one-to-one interview if they feel more comfortable sharing their views in this way.

**Topic Areas**

The meeting will be semi-structured in nature to allow exploration of key areas relating to stakeholders’ experiences of participating in a co-production process as well as explore new areas identified by participants. An outline of the key areas to be explored and example questions are provided below. The areas will be further informed by the observations of the stakeholder meetings. The exact nature and wording of the questions will vary to allow the interview to be guided by the stakeholders and explore areas important to them.

**Introductory Questions**

The interview will start with some broad questions to introduce the interview and encourage the participant to discuss their view. These questions will include:

- Can you tell me about your role in the stakeholder group?
- Please tell me about how you found the experience of working together to develop a new approach for falls management in care homes

**Key Themes**

The interview will focus on the key areas of interest below based on the principles of co-production (1,2):

**Sharing of power**

**Including all perspectives and skills**

**Respecting and valuing knowledge**

**Reciprocity**

**Building and maintaining relationships**

Based on these areas of interest, additional questions may include:

**How were decisions made during the process?**

**How engaged did you feel in the process?**

**How confident did you feel to share your views?**

**How confident did you feel to challenge the views of others in the group?**

**How satisfied did you feel with the approach that the group decided on?**

**How respected and valued did you feel?**

**Were there any opportunities for personal growth and development? (If so, what were these?)**

**How did relationships develop between group members over time?**

**What were the challenges of working together as equal stakeholders?**

**What helped you to work together as equal stakeholders?**

**What could have been done differently?**

**Were there any benefits of working together? (If so, what were these?)**

References

1. National Institute for Health Research. Guidance on co-producing a research project. Available from: <https://www.learningforinvolvement.org.uk/?opportunity=nihr-guidance-on-co-producing-a-research-project> [Accessed 8^th^ Feb 2022]
2. Leask CF, Sandlund M, Skelton DA, Altenburg TM, Cardon G, Chinapaw M, et al. Framework, principles and recommendations for utilising participatory methodologies in the co-creation and evaluation of public health interventions. Research Involvement and Engagement. 2019;5:2.

**Meeting Script**

Thank you for agreeing to take part in the reflection meeting today to talk about your experiences of taking part in co-production meetings to develop a new way of managing falls in care homes.

Taking part in the interview is completely voluntary. There are no right or wrong answers, we are interested in your views and experiences of working together as equal stakeholders in the meetings you attended over the last year.

We anticipate that the meeting today will last no longer than an hour but please stop me at any point if you would like a break or would like to stop the meeting.

We will [record the interview with your consent/make written notes along the way] and with your permission anonymous quotes may be used in reports to share the study findings.

To maintain confidentiality, we ask that you please do not mention any personal information or names that may identify specific staff or other residents who are not stakeholders in this group.

The content of the interview will remain confidential and only used for the purpose of this research however if we consider anything you disclose during the interview requires escalation as it relates to harm of another person we will be obliged under our professional code of conduct to follow safeguarding procedures of the University of Nottingham:

We do not anticipate that the meeting will be distressing and please only answer and talk about things that you feel comfortable with. If you do feel uncomfortable at any time, please just let us know and we can stop the meeting at any point.

Is there anything you would like to ask us at this point before we start the meeting? Are you happy to continue with the meeting?
